# The eye-voice span during reading aloud

**DOI:** 10.3389/fpsyg.2015.01432

**Published:** 2015-09-24

**Authors:** Jochen Laubrock, Reinhold Kliegl

**Affiliations:** Department of Psychology, University of Potsdam, PotsdamGermany

**Keywords:** reading, eye movements, eye-voice span, synchronization, working memory updating, psychologinguistics

## Abstract

Although eye movements during reading are modulated by cognitive processing demands, they also reflect visual sampling of the input, and possibly preparation of output for speech or the inner voice. By simultaneously recording eye movements and the voice during reading aloud, we obtained an output measure that constrains the length of time spent on cognitive processing. Here we investigate the dynamics of the eye-voice span (EVS), the distance between eye and voice. We show that the EVS is regulated immediately during fixation of a word by either increasing fixation duration or programming a regressive eye movement against the reading direction. EVS size at the beginning of a fixation was positively correlated with the likelihood of regressions and refixations. Regression probability was further increased if the EVS was still large at the end of a fixation: if adjustment of fixation duration did not sufficiently reduce the EVS during a fixation, then a regression rather than a refixation followed with high probability. We further show that the EVS can help understand cognitive influences on fixation duration during reading: in mixed model analyses, the EVS was a stronger predictor of fixation durations than either word frequency or word length. The EVS modulated the influence of several other predictors on single fixation durations (SFDs). For example, word-N frequency effects were larger with a large EVS, especially when word N-1 frequency was low. Finally, a comparison of SFDs during oral and silent reading showed that reading is governed by similar principles in both reading modes, although EVS maintenance and articulatory processing also cause some differences. In summary, the EVS is regulated by adjusting fixation duration and/or by programming a regressive eye movement when the EVS gets too large. Overall, the EVS appears to be directly related to updating of the working memory buffer during reading.

## Introduction

The pattern of fixations and saccades during reading is arguably one of the most practiced and fastest motor activities humans routinely perform. Eye movements during silent reading are clearly affected by cognitive processing. Both low-level visuo-motor factors and high-level comprehension processes co-determine where the eyes land within a word during reading (see Rayner, 1998, 2009, for reviews). Cognitive modulation of oculomotor control has been incorporated in all successful computational models of eye movements during reading, such as SWIFT (Engbert et al., 2002, 2005), EZ-reader (Reichle et al., 1998, 2003), or Glenmore (Reilly and Radach, 2006). However, almost all of the data on which these models are based originates from studies examining silent reading. Here we argue that, by measuring the dynamics between eyes and voice during oral reading [i.e., differences between the fixated and pronounced words related to processing difficulty at a given point in time; eye-voice span (EVS)], we obtain information about limits of phonological representations of words in working memory (Inhoff et al., 2004), episodic buffer (Baddeley, 2000), or long-term working memory (Ericsson and Kintsch, 1995), available for cognitive processing of the text. Fixation location approximately tells us which input is processed at any point in time, taking into account the fact that the perceptual span during reading has a maximum extent of 10–15 characters to the right of fixation (Rayner, 1975). Articulatory output of a word presumably tells us that it no longer needs to be buffered in working memory. Note that these limits are obtained during a continuous updating of working memory. Indeed, the regulation of the EVS by local processing difficulty may be the most direct measure of limits associated with these constructs. It may also provide additional constraints for computational models of eye-movement control during reading.

Silent reading is a fairly recent cultural invention, at least in the West, where it was introduced only around the 8th century, following the introduction of word spaces (Manguel, 1996). Even though there are reported instances of reading silently, reading aloud was the default in classical antiquity. Similarly, reading aloud precedes silent reading in individual development, for example, in primary school education. Thus, in addition to developing a mental model of the text, a major goal of the reading process is to prepare the words for pronunciation. Indeed, there is evidence that subvocalization takes place even during silent reading and typically occurs during fixation of the subsequent word (Inhoff et al., 2004; Eiter and Inhoff, 2010; Yan et al., 2014a).

Given the importance of oral reading, the lack of data on the coordination of eye and voice during oral reading is surprising. Most of the available data appear to originate with Buswell’s (1920, 1922 seminal work using an early eye tracker (see also Tiffin, 1934, for an early approach at simultaneous recording). Buswell (1920) found that the pattern of eye movements during oral reading, just like the pattern during silent reading, consists of forward saccades, regressions, refixations, and word skippings. More recent research supports the view that eye movements during silent and oral reading are qualitatively similar, although there are also a number of consistently reported quantitative differences. Due to the additional articulatory demands, the average fixation duration is about 50 ms longer in oral reading, the average saccade length is shorter, and there are more regressions (Rayner et al., 2012, p. 92; Inhoff and Radach, 2014). However, the correlation between eye-movement measures obtained during silent and oral reading is high (Anderson and Swanson, 1937). In essence this suggests that oral reading processes may be essentially the same as silent reading processes, but that readers don’t want the eyes to go too far ahead of the voice.

Parafoveal processing of upcoming text is important for efficient silent reading (e.g., Sperlich et al., 2015). Interestingly, although parafoveal processing also plays a role in oral reading, the size of the perceptual span is smaller in this mode, possibly related to the overall decrease in saccade size, (Ashby et al., 2012) or the later use of parafoveally extracted information (Inhoff and Radach, 2014). Thus although more time is available due to the longer fixations in oral reading, apparently this time is not used in the same way for parafoveal preprocessing. Nevertheless, given that parafoveal processing plays a role in silent reading, the spatial region of information extraction and cognitive processing is somewhat larger than the EVS.

Buswell (1920) defined EVS as the distance that the eye is ahead of the voice during reading aloud. He reported the EVS to be on the order of 15 letters (or two to three words) for college students and as increasing over the course of high-school education (Buswell, 1920, Table 1). Buswell also reported that the EVS is sensitive to local processing difficulty, e.g., he found an increased number of regressions (saccades against the reading direction) following a large EVS (see also Fairbanks, 1937). However, he did not have available the rich set of tools that statistics and psycholinguistics provide us with today. These allow us to examine influences of linguistic word properties (e.g., word length, frequency, and predictability) of the currently fixated word or of its neighbors on eye-movement measures of the currently fixated or the currently spoken word. Linear mixed models (LMMs) allow us to evaluate the degree of parallel processing. For example, we can re-evaluate Buswell’s hypothesis that the EVS might be responsible for long fixation durations—a hypothesis he could not confirm with his analysis methods (Buswell, 1920, pp. 80–81).

The empirical database on the EVS during reading aloud is very sparse, and most published articles after Buswell used a rather imprecise oﬄine method, that is without recording of eye movements (e.g., Levin and Buckler-Addis, 1979). The oﬄine method works by switching off the light during reading of a sentence and counting how many words can be articulated after the light was off. Obviously, this “off-line EVS” not only includes parafoveal preview and guessing, but may also depend on task-dependent strategies such as looking at the final part of the sentence before starting to read aloud. For these reasons, the oﬄine EVS typically ranges from 6 to 10 words and, to anticipate one of our results, grossly overestimates the EVS measured with eyetracking equipment. Using an eyetracker, Inhoff et al. (2011) determined the temporal EVS, that is the average time the voice trails behind the eyes. They found an average temporal EVS of about 500 ms, which is in good agreement with Buswell (1920), but certainly too short to process 6–10 words, given an average fixation duration of 250 ms. In the most recent study, De Luca et al. (2013) reported a spatial EVS of about 13.8 letters for normal and of 8.4 letters for dyslexic readers.

What does the EVS measure? Although it is possible that synchronization of the eyes with the speed of articulation is attempted for no particular reason, the EVS is more likely related to updating of working memory. During the time between visual input and speech output, the written text is transformed into a phonological code, which is then buffered in the phonological loop (Baddeley and Hitch, 1974). The need for translation into a phonological code arises from the fact that purely visual short-term memory decays very quickly (Sperling, 1960). Buffering is necessary because the articulatory motor system is just too slow to produce understandable speech at the maximum rate of visual decoding and grapheme-to-phoneme conversion. In support of this view, Pan et al. (2013) found that the EVS in a RAN task correlated with naming speed only when highly familiar and practiced symbols (digits) were named automatically, but not with naming of less well-practiced items with identical articulatory demands (number of dots on a dice). Moreover, dyslexic readers did not exhibit this correlation between EVS and automatic naming of digits, suggesting that a larger EVS is indicative of buffering of material that can be rapidly decoded and translated from graphemic input into a phonological code. Buffering is followed by selection of and commitment to a single phonological code in order to conduct explicit programming for the articulatory response (Jones et al., 2015). Thus dyslexic readers also exhibit a temporal EVS delay on RAN, which is specific to this measure, i.e., no analogous deficit appears in gaze duration (GD; Jones et al., 2013).

Such a first-in-first-out buffer is conceptualized with a finite and rather limited capacity, that is, it cannot sample input infinitely when no output occurs. In general, as we don’t appear to use visual short-term memory for buffering of text, most of the buffering during oral reading is probably on the phonological side of the translation, but before the actual articulatory motor processes start. This is compatible with estimates of the inner voice during silent reading: phonological codes appear to be activated for most words we read and this phonological information is held in working memory and is used to comprehend text (Rayner et al., 2012, chap. 7). These phonological codes lag behind the eyes in reading. The phonological buffer in the Baddeley and Hitch (1974) working-memory model has a special capacity for temporal order information. Thus, one important function of phonological codes is to provide access to the order in which words were read.

Synchronized recordings of eye movements and other motor activity are occasionally reported from other domains (Land and Tatler, 2009, for an overview); for example there are several reports of the eye-hand span during piano playing (Truitt et al., 1997; Furneaux and Land, 1999), writing (Almargot et al., 2007), typewriting (Butsch, 1932; Inhoff et al., 1986; Inhoff and Wang, 1992; Inhoff and Gordon, 1998), or performing sports (Land and Furneaux, 1997; Land and McLeod, 2000). One general finding emerging from these studies is that the eye-hand span increases with expertise if measured in units of information (letters or notes), whereas it appears to be fairly constant at around one second if measured in units of time (e.g., Butsch, 1932; Furneaux and Land, 1999). Although these data are only indirectly related to oral reading because of obvious differences in input information and effector system, they are similar in the need to coordinate fast eye movements and a much slower motor system. In particular, working memory buffering is also needed for other forms of output, but may use different codes depending on the output demands.

The aims of the current study are twofold. First, we measure visual sampling of the input and oral output simultaneously to obtain a precise estimate of cognitive processing times during oral reading., These data yield a description of the EVS that allows us to evaluate Buswell’s (1920) findings with state-of-the art equipment. Second, we investigate the dynamics of the EVS during reading aloud with LMMs for statistical inference and with reference to the possible role of working memory. In perspective, these analyses are to provide constraints for computational models of eye movement control during reading.

Arguably, during silent reading there are well-documented effects of neighboring words on fixation duration (Kliegl et al., 2006; Kliegl, 2007; Wotschack and Kliegl, 2013; but see Rayner et al., 2007). For example, Kliegl et al. (2006) examined the effect of word frequency of the current, past, and upcoming word on current fixation duration during the reading of German sentences. They reported that the negative linear influence of word frequency of the currently fixated word was weaker than that of the word frequency of its left neighbor, indicating that lagged cognitive processing can directly influence saccade programming (see also Rayner and Duffy, 1986). There was also a weak, but significant negative effect of the word frequency of the right neighbor. Moreover, in the same analyses, the predictability of the upcoming word prolonged fixation durations, as indicated by a significant *positive* effect of the predictability of its right neighbor, suggesting that memory retrieval of the right-parafoveal word is attempted when it is likely to be successful. These effects were obtained across nine independent samples of readers (Kliegl, 2007).

Experimental evidence for preprocessing of the parafoveal word to the right also comes from studies using the gaze-contingent display-change paradigm (Rayner, 1975), in which a preview is replaced by a target word during a saccade to the target; preview benefit is the reduction of target fixation duration as a function of the relatedness of the preview relative to a non-word or unrelated preview word. Orthographic and phonological information has long been known to produce preview benefits (e.g., Rayner, 1978; Rayner et al., 1978; Pollatsek et al., 1992; Henderson et al., 1995), and although overall the data are not completely clear (Rayner, 2009), evidence is accumulating that semantic relatedness can also result in preview benefit (Yan et al., 2009; Hohenstein et al., 2010; Laubrock and Hohenstein, 2012; Schotter, 2013).

In summary, during a fixation on a word, processing of the last and of the upcoming word as well as predictive processes are simultaneously ongoing. Given that not only properties of the current word, but also those of its neighbors influence a fixation duration, the question arises to what extent they also affect the EVS. Conversely, how does the EVS affect the where and when of eye movement programming? Having access to an explicit measure of the EVS allows us to answer these questions in detail. The goal of the present work is to present a rich description of the EVS, its relation to eye-movement behavior and to cognitive demands. In perspective, we aim for a novel, on-line characterization of the working memory buffer during actual reading that we hope stimulates and constrains further modeling attempts.

## Materials and Methods

### Participants

Thirty-two subjects (12 males, 20 females) received 7 € or course credit for participating in an oral experiment lasting approximately 40 min. Their mean age was 18 years (*SD* = 1.5 years, range = 16–24 years). An additional 31 subjects (12 males, 19 females; mean age 19 years, *SD* = 1.4 years, range = 16–24 years) read the same sentences in a silent reading experiment. All subjects had normal or corrected-to-normal vision. Experiments comply with the June 1964 Declaration of Helsinki (entitled “Ethical Principles for Medical Research Involving Human Subjects”), as last revised, concluded by the World Medical Association. Our eye-tracking research has been approved by Ethikkommission der DGPs (Registriernummer: JKRKRE19092006DGPS).

### Apparatus and Material

Sentences were presented on a 22″ Iiyama Vision Master Pro 514 CRT monitor with a resolution of 1280 × 960 pixels controlled by a custom C++ program running on a standard PC. Voice was recorded to hard disk using a Sennheiser K6 series condensator microphone connected to an ASIO compatible SoundBlaster Audigy sound card inside the PC, ensuring a fixed audio latency of 5 ms. Eye movements were registered using the Eyelink 1000 tower mount (SR Research, Ottawa, ON, Canada). The head was stabilized and a viewing distance of 60 cm was assured with a headrest, but the usual additional chinrest was removed to allow for easy articulation. Eye movements and voice protocols were synchronized by sending trigger signals to the eye tracker at the beginning and end of each sound recording, which were recorded in tracker time in the series of eye tracker time stamps and later adjusted for the audio output delay.

The experimental material was the Potsdam Sentence Corpus 2 (PSC2), consisting of 144 simple, declarative German sentences taken from various newspapers (Poltrock, unpublished Diploma thesis). Word length ranged from 2 to 13 letters (*M* = 5.26, *SD* = 2.59 letters), sentence length ranged from 7 to 13 words (*M* = 8.54, *SD* = 1.44) and from 34 to 84 letters (*M* = 54.58, *SD* = 10.67). Word frequency information for the 1230 words was obtained from the DWDS/dlexdb corpus (Heister et al., 2011) based on ca. 120 Million entries. Median word frequency was 234.2 per Million, and the range was from 0.008 to 26530 per Million (for “Geplä nkels” and “der”, respectively). Incremental cloze predictabilities were collected from different 283 participants generating more than 85,000 predictions (mean N of predictions per word 69.6, range from 57 to 84) using an internet-based questionnaire, combined with an ipod lottery to increase motivation. The mean predictability over words in the corpus was 0.188, and the median predictability was 0.042; about 1/3 of all words were completely unpredictable. As usual in single-sentence material, predictability in the PSC2 increases with position of word in the sentence (e.g., mean predictability of 0.063 and 0.435 for sentence-initial and sentence-final words, respectively).

### Procedure

The 144 experimental sentences were read in random order after six initial training sentences used to familiarize the participants with the task and to adjust the volume/gain setting of the microphone. One sentence was presented per trial, vertically centered on the screen, in black on a white background, using a fixed-width Courier New font with a font size of 24 points. A letter subtended 14 pixels or 0.45° of visual angle horizontally. A trial started with a drift correction in the screen center (standard drift correction target), followed by presentation of a gaze-contingent sentence trigger target 18.1° to the left of the screen center, followed by presentation of the sentence. The sentence was only revealed after the gaze-contingent trigger had been fixated for at least 50 ms. Visual properties of the sentence trigger target were identical to those of the drift correction target. Sentences were aligned with the center of the first word positioned slightly to the right of the sentence trigger target; so that the gaze was initially positioned at the first word’s optimal viewing position. Sentence presentation ended when subjects fixated a point in the lower right screen corner. To ensure that subjects read the sentences and not just moved their eyes, a randomly determined third of sentences were followed by an easy comprehension question, requiring a three-alternative choice response.

The eye tracker was calibrated at the beginning of the experiment and after every 36th trial or whenever calibration was bad. Bad calibrations were detected at the beginning of each trial: when the gaze was not detected within an area of 1° centered on the sentence trigger target within 1 s from the start of its presentation, a re-calibration was automatically scheduled. A trial ended when subjects fixated another gaze-contingent trigger (150 × 150 pixels square) in the bottom right corner of the screen for at least 50 ms, which was visually represented by a 5-×-5 pixel in its center.

### Data Analysis

#### Eye Movement Recordings

The horizontal position of the gaze was mapped to letter positions, and standard measures were determined such as first-fixation duration (FFD; duration of the first fixation on a word in firstpass reading), single fixation duration (SFD; duration of fixations on words that received exactly one first-pass fixation), GD (sum of all first-pass fixations) as well as skipping refixation, regression, and single-fixation probabilities. Trials with eye blinks were removed from the analysis. Also data from the first and last words of each sentence were not included in the analysis.

#### Voice Recordings

A Praat (Boersma, 2001; Boersma and Weenink, 2010) script was prepared that looped over subjects and sentences and presented each sentence (divided into words) together with its associated sound recording, showing a representation of the waveform together with a spectrogram, formants, and intensity and pitch contours. The script attempted to locate the beginning and end of spoken parts by crossings of an intensity threshold, and initially distributed word boundaries across the spoken part in proportion to word length. Human scorers then manually dragged word boundaries to the subjective real boundary locations by repeatedly listening to stretches of the speech signal. Several zoom levels were available, and scorers were instructed to zoom in so far that only the word in question and its immediate neighbors were visible (and audible) for the ultimate adjustment. In the case of ambiguous boundaries due to co-articulation, scorers were instructed to locate the boundary in the middle of such ambiguous stretches^1^. Only articulated word durations from sentences that were read without error were used in further analyses.

#### Eye-Voice Span

The 86% of sentences (3938 out of 4608) with correct articulation and without eye blinks were used in analyses of the EVS. The EVS can be defined in either temporal or spatial units, or either relative to the fixated or the articulated word. As temporal measures, we calculated the time difference in milliseconds to articulation onset at the beginning of the first fixation on a word (termed *onset-EVS* below) and at the end of the last fixation on a word (*offset-EVS*). As spatial measures, we calculated the distance in letters of the currently articulated letter relative to each fixation onset and offset.

Labeling word boundaries in the auditory signal is like sampling the signal only at word boundaries. However, the eye and voice are to a certain degree independent of each other, that is fixations usually start during the pronunciation of a word. In an attempt to increase the precision of the position of the voice at fixation onset, we made use of the very high linear correlation between articulated word times and word length in German (*r* = 0.86 in the present data). Specifically, we linearly interpolated letters by assuming that the per-letter duration is given by the word’s articulated duration divided by its number of letters to estimate the proportion of a word that was spoken at fixation onset. For most analyses reported below, the spatial distance in letters at first-fixation onset or offset will be used.

#### (Generalized) Linear Mixed Models

Analyses were performed with the R statistical computing environment (R Development Core Team, 2015) and the packages lme4 (Bates et al., 2015b) and remef (Hohenstein and Kliegl, 2015), using a LMM approach that allows to investigate experimental effects with statistical control due to differences between subjects and sentences as random factors (Bates et al., 2015a). We used two GLMMs and two LMMs. With the two GLMMs we modeled regressive and refixation saccades as a function of either onset EVS and the change in EVS (from onset-EVS to offset-EVS) during a fixation using the logit link, with statistical control for differences between participants and sentences. With the two LMMs we modeled SFDs; to achieve normally distributed residuals, SFDs were log transformed. Both models used the covariates reported in Kliegl et al. (2006) with nine word and three oculomotor variables as a starting point (see Results for details). These covariates are not necessarily in a strict linear relation with the dependent variable. Therefore, to guard against overlooking an important non-linear contribution, we modeled these covariates with quadratic polynomials, except frequency of the fixated word for which we specified a cubic trend (see Heister et al., 2012). To the first LMM, we added EVS (a linear within-subject covariate) and its interactions with all the other covariates as additional fixed effects. Analogously, we added reading condition (oral vs. silent; a between-subject fixed factor) and its interactions with the other covariates. Thus, the two LMMs were of equal complexity. Moreover, for all models we determined significant variance components for experimental effects and associated correlation parameters. In principle, there is no upper limit to model complexity with 12 quadratic (or higher-order) covariates. Therefore, we built the LMM with the constraint that the model was not overparameterized, following recommendations and procedures in Hohenstein and Kliegl (2014) and Bates et al. (2015b). Data, scripts, and results of all analyses are available as a supplement at Rpubs.com.

## Results

General descriptive statistics relating to eye movements and articulation during oral reading are summarized in **Table 1**. For comparison we include also eye movement data from a new sample of 31 readers who read the same material silently. The comprehension questions were accurately answered in both reading modes, with mean accuracies of 97.7% (range 94–100%) for oral and 97.4% (range 94–100%) for silent reading. Fixation durations were longer and saccades were shorter during oral than during silent reading. The probability of refixating a word was higher, whereas the probabilities of word skipping and of regressions were lower in oral than in silent reading. The average spoken word duration in oral reading was similar to the average GD. Notably, the time till pronunciation of the first word was about the duration of three spoken words, suggesting that the eye initially gets a head start before articulation of the sentence starts.

**Table 1:** Descriptive statististics for oral and silent reading.

	Oral		Silent	
	*Mean*	*SD*	*Mean*	*SD*
Fixation duration [ms]	253	96	209	81
First fixation duration [ms]	262	96	213	81
Single fixation duration [ms]	273	99	216	82
Gaze duration [ms]	334	162	247	124
Total viewing time [ms]	362	187	288	173
Saccade length [letters]	5.9	2.6	7.0	3.1
Skipping probability	0.14	0.35	0.21	0.41
Single fixation probability	0.51	0.50	0.59	0.49
Refixation probability	0.18	0.38	0.11	0.31
Regression probability	0.06	0.23	0.10	0.30
Time to first-word pronunciation [ms]	877	191		
Spoken word duration [ms]	293	150		

In the following we focus on the dynamic relation of eye and voice. The presentation of results is organized as follows. In the first sections the focus is on active control of EVS by regression, refixation, and fixation durations. The final section informs about whether previously reported effects of distributed processing of words in the perceptual span on fixations during silent reading are also observed during oral reading.

### Eye-Voice Spans

**FIGURE 1:**
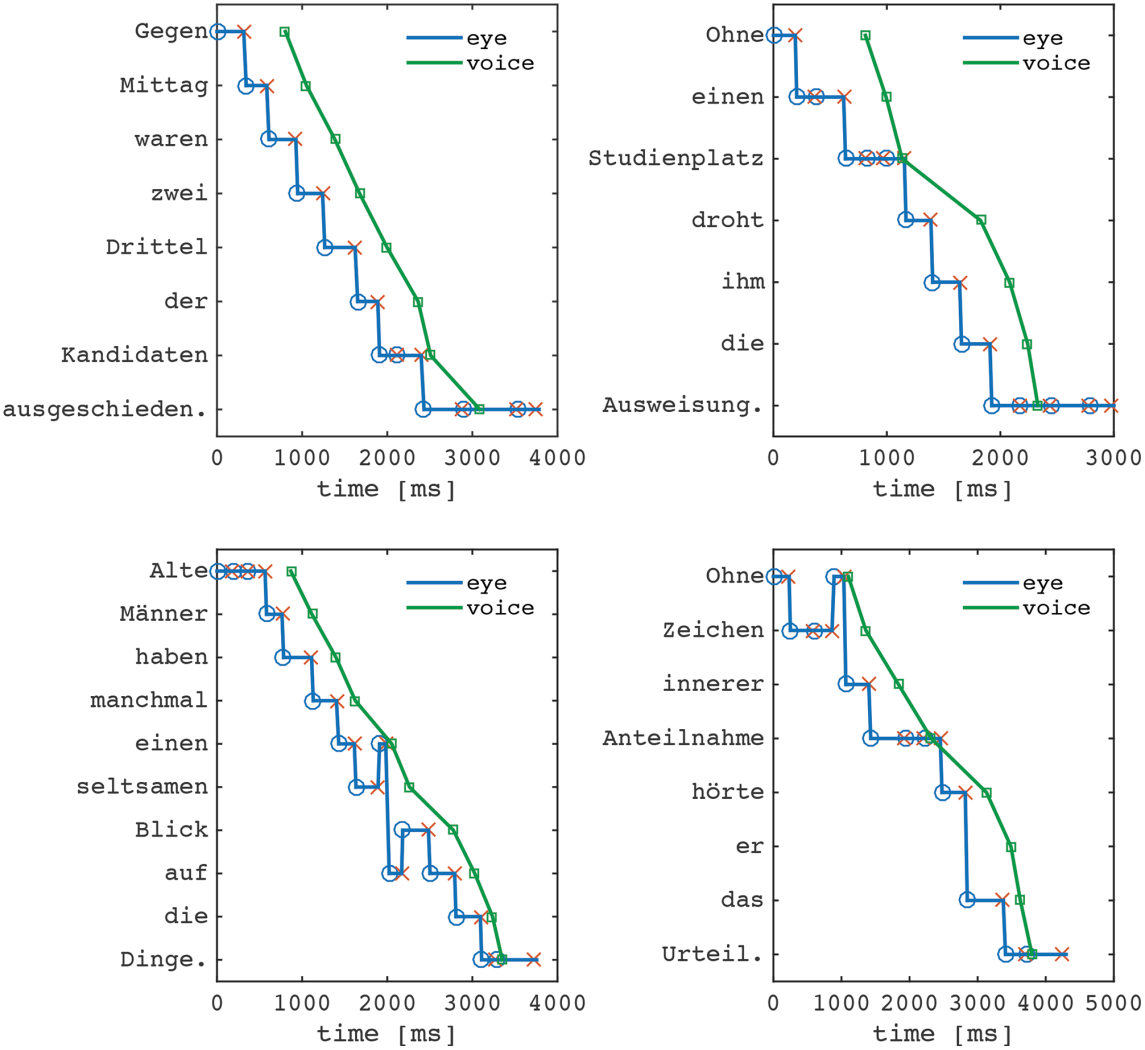
**Examples of eye and voice positions over time during reading of three different sentences.** The blue trace shows the eye position, with circles marking fixation onsets and Xs marking fixation offsets. The green line shows the onset times of each word’s pronunciation. See text for details.

The signature marker of oral reading is the EVS, which can be measured with respect to the temporal or the spatial distance between eye and voice. We illustrate these concepts with three examples. Each panel in **Figure 1** shows the traces of the eye (blue line) and the voice (green line) over time during the reading of a sentence. In the top left panel the eye leads the voice by a fairly constant time or distance throughout the sentence. In the top right panel, the EVS all but vanishes during refixations of the word “Studienplatz.” In the bottom left panel, the eye regresses back twice to previous words to wait for the voice to catch up, followed by the eye jumping ahead of the voice again to ensure a distance similar to the one before the regression. Arguably, the latter two cases represent prototypes of how eye and voice take care of a local disturbance. Often this is due to a particularly difficult word, like in the refixations example where, in a way, the difficult word serves as a point of synchronization. The determiner “einen,” on the other hand, is unlikely to cause processing difficulties in normal reading, possibly the function of the regression is to reduce the distance between eye and voice. In the bottom right panel, finally, regressions and refixations are displayed, and a particular pattern appears at the beginning of the sentence, where the eye initially scouts ahead, and makes a regression to the beginning word just before the voice starts pronouncing it. This sentence-initial pattern that looks like an initial re-synchronization to maintain a manageable buffer size was quite typical.

#### Temporal EVS

**FIGURE 2:**
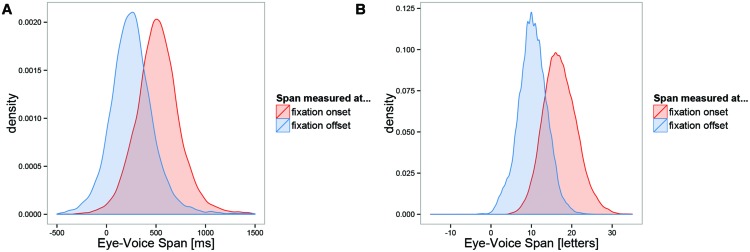
**Distribution of the eye-voice span (EVS). (A)** Time from onset or offset of the first fixation on a word until beginning of pronunciation of the word, **(B)** spatial distance in letters between position of the eye and (interpolated) position of the voice at fixation onset or offset. Positive numbers indicate that the eye is ahead of the voice.

The temporal EVS distributions are displayed in the left panel of **Figure 2**. The distribution of the EVS in milliseconds from the beginning of the first fixation on a word to the onset of its pronunciation was nearly symmetric, with a mean of 561 ms and a standard deviation of 230 ms (**Figure 2**, right distribution in left panel). In contrast to most other measures during reading, the interindividual variability in temporal EVS (*SD* = 73 ms) was smaller than the intraindividual variability (*SD* = 218 ms). The mean EVS per subject ranged from 428 to 781 ms in our sample. Obviously, during oral-reading fixations the voice is able to catch up with the eyes. Consequently, the temporal EVS from the end of the last fixation on a word to the onset of its pronunciation was much shorter with a mean of 254 ms and a standard deviation of 216 ms (**Figure 2**, left distribution in left panel). The standard deviations of the onset and offset distributions were not significantly different; Levene’s test, *F* = 2.66, *p* = 0.103.

#### Spatial EVS

The spatial EVS distributions are displayed in the right panel of **Figure 2**. The distance in letters between the position of the eye and the position of the voice was estimated at each fixation onset after articulation of the words had started. Like the temporal EVS distribution, the spatial EVS distribution was nearly symmetric and showed considerable variability. The distribution at first fixation onset had a mean of 16.2 letters (*SD* = 5.2 letters). The interindividual variability (*SD* = 1.5 letters) was smaller than the intraindividual variability (*SD* = 4.9 letters). At last fixation offset, the eye was still 9.7 letters ahead of the voice (*SD* = 3.6 letters). Thus, during a fixation the spatial EVS was reduced on average by 6.5 letters (which is very close to the average saccade size); moreover, this reduction in spatial EVS went along with a significant reduction of its standard deviation; Levene’s test, *F* = 797, *p <* 0.001. We interpret these results as evidence for active control of spatial rather than temporal EVS.

### Eye-Voice Span as Predictor of Eye-Movement Control

A dominant goal of oral reading is to maintain a steady pace, modulated only for various prosodic effects. The observation that fixation durations are locally adjusted to keep the EVS at fixation offset at a fairly constant level of about 10 characters reflects this regulation. In this section we analyze by which means active control of spatial EVS is achieved. Specifically we show that at a given point in time the EVS is predictive of (1) regressions, (2) refixations, and (3) fixation durations that are followed by a forward saccade. Note that with this definition we analyze three non-overlapping sets of fixations and their associated EVSs from reading the same sentences.

#### Spatial EVS Predicts Regression and Refixation Probabilities

Moving beyond anecdotal evidence and descriptives, we demonstrate regulation with analyses of regression and refixation probabilities as a function of EVS at the beginning and at the end of a fixation. Effects were tested with two GLMMs using the logit link function to predict binomial responses (either refixations or regressions) with EVS at onset and the difference between onset-EVS and offset-EVS as predictors, including both linear and quadratic trends.

**FIGURE 3:**
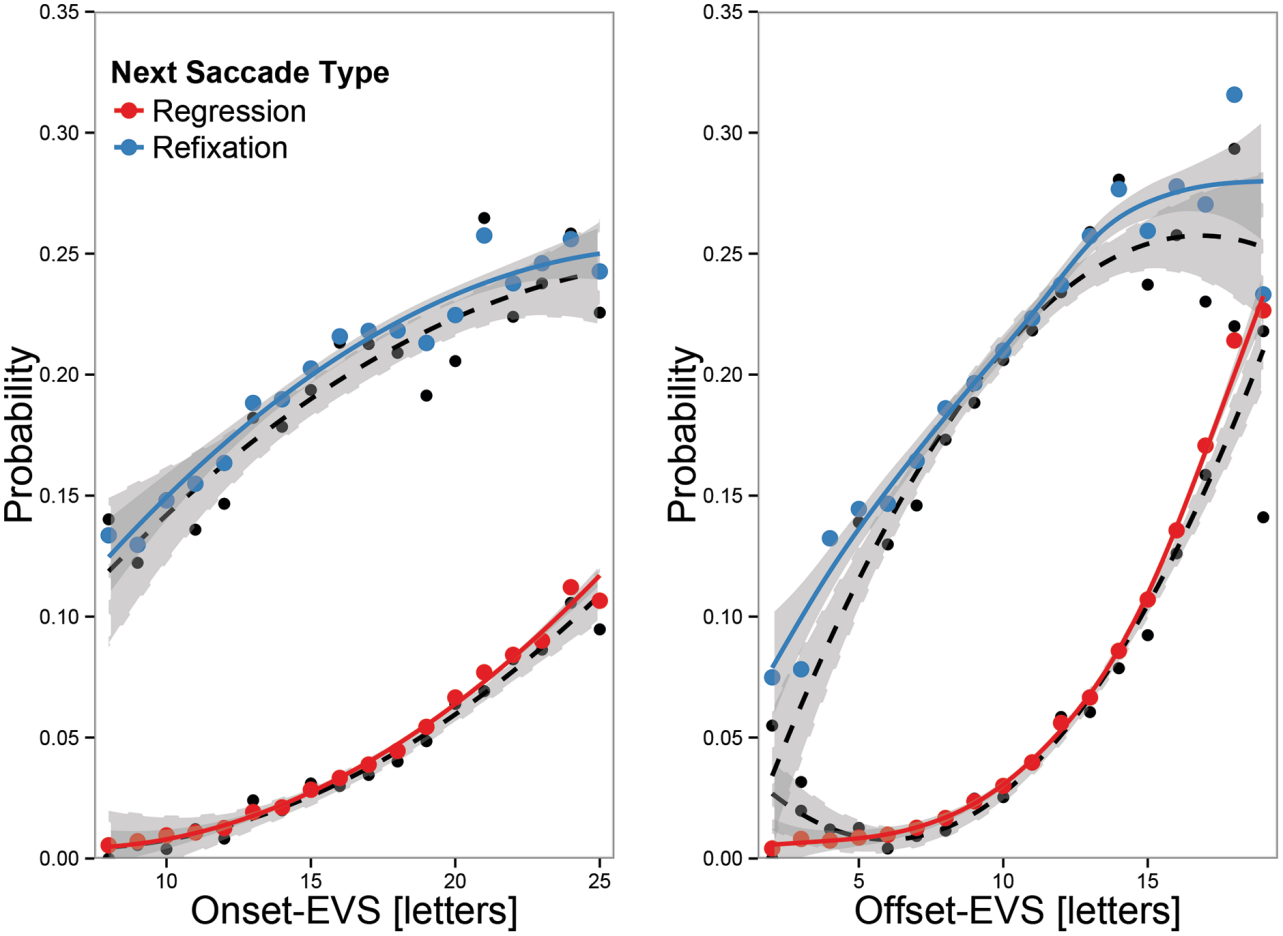
**Regression and refixation probabilities as function of EVS at fixation onset **(left)** and offset (right).** Black dots represent overall means, and colored dots predicted means, adjusted for random effects. The lines represent second-order polynomial regression fits (black dotted) or GLMM fits (colored, solid). EVS at fixation onset is already predictive of an upcoming regression or refixations, but offset-EVS is more predictive. When EVS was large at offset, there was a high likelihood of making a regression.

The left panel of **Figure 3** shows the key results for regression and refixation probabilities as a function of the EVS at fixation onset. Both probabilities increased with an increase in EVS, suggesting that it is often determined already at the onset of a fixation whether a halt or a regressive eye movement will be programmed. **Table 2** shows that for both refixations and regressions, there were purely linear effects on the logit scale, indicating that the odds of making a regression or refixation increase with every character increase in the onset-EVS.

**Table 2:** Estimates of GLMMs for regressions (upper part) and refixations (lower part) as a function of the Eye-Voice-Span.

Regressions							
**Fixed effects**							
			**Estimate**	***SE***	***z* value**	***p***	**Sig**
		(Intercept)	-3.82	0.16	-24.36	<0.001	***
		Onset-EVS, linear	146.60	8.25	17.78	<0.001	***
		Onset-EVS, quadratic	5.89	6.29	0.94	0.35	
		ΔEVS, linear	97.68	7.06	13.83	<0.001	***
		ΔEVS, quadratic	6.47	6.23	1.04	0.3	

**Random effects**							
		**Groups name**	**Variance**	***SD***			

		Sn	(Intercept)	0.31	0.56		
		Id	(Intercept)	0.58	0.76		
		Number of obs: 16451, groups: sn, 144; id, 32					

**Refixations**							

**Fixed effects**							
			**Estimate**	***SE***	***z* value**	***p***	

		(Intercept)	-1.59	0.10	-16.64	<0.001	***
		Onset-EVS, linear	60.44	3.38	17.89	<0.001	***
		Onset-EVS, quadratic	-2.39	2.83	-0.85	0.40	
		ΔEVS, linear	49.86	3.32	15.02	<0.001	***
		ΔEVS, quadratic	-10.92	3.04	-3.59	<0.001	***

**Random effects**							
		**Groups name**	**Variance**	***SD***			

		Sn	(Intercept)	0.28	0.53		
		Id	(Intercept)	0.21	0.46		
		Number of obs: 16451, groups: sn, 144; id, 32					

*ΔEVS indicates the effect of the difference of offset-EVS minus onset-EVS.*

The right panel of **Figure 3** shows that the correlation between the offset-EVS and regression and refixations probabilities was considerably stronger at fixation offset than at fixation onset. This is captured by a significant coefficient for the ΔEVS-effects in **Table 2**. For both regressions and refixations, there was a strong increase in the linear effects. Additionally, there was a negative quadratic trend for refixations, meaning that when offset-EVS was very large, the likelihood of refixating increased no further; so that when offset-EVS was large, the probability of making a regression exceeded the refixations probability (the apparent positive quadratic trend for regressions was linear on the logit scale, indicating that with every character increase in the EVS, there is a proportional increase in the odds of making a regression). The fact that offset-EVS is more strongly related to regression behavior than onset-EVS suggests that the control of fixation durations is sometimes successful in decreasing the EVS.

In summary, the EVS is regulated by programming a refixation or a regression when the EVS gets too large. Whether a refixation or a regression is programmed is related to the size of the EVS at fixation offset: the likelihood of making a regression strongly increases with every additional character of EVS, whereas the likelihood of making a refixation initially increases, but then drops again for large EVS, for which regressions are the rule. The increase in regression or refixations probabilities with offset-EVS was larger than with onset-EVS. Taken together, this suggests that regressions or refixations are programmed when the control of fixation duration is not sufficient in down-regulating the EVS.

#### Spatial Onset EVS Predicts Fixation Durations

##### Main effect of EVS

**FIGURE 4:**
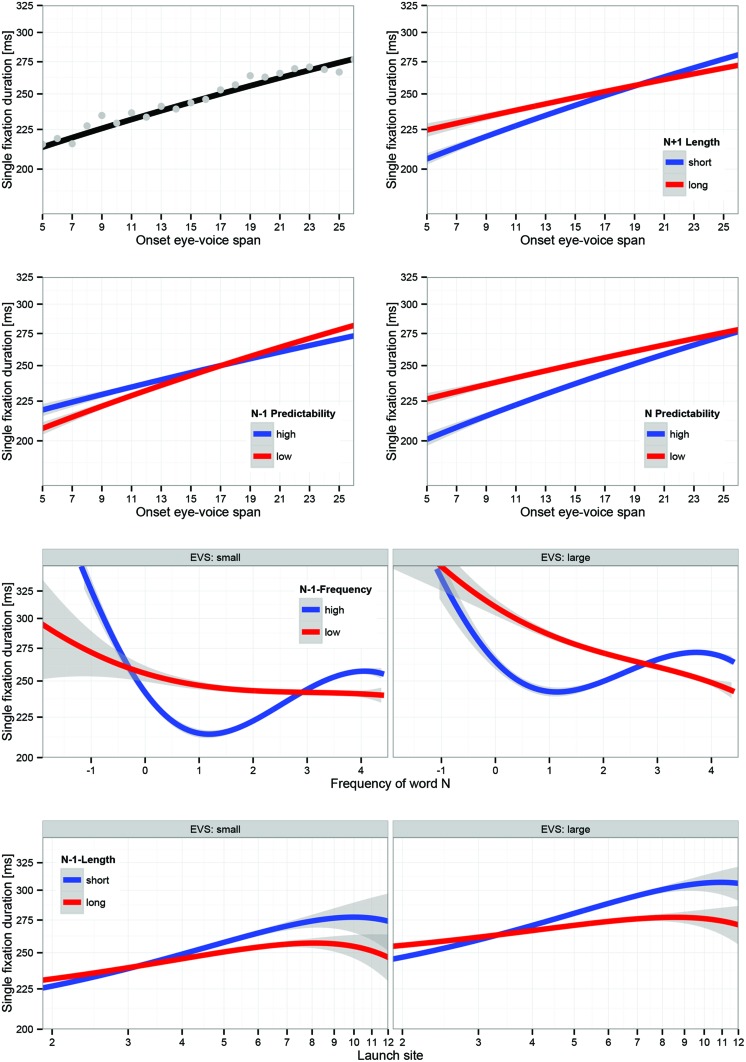
**Visualization of LMM estimates of main effect of onset EVS and three EVS-related interactions; LMM used three continuous covariates. Top left** : main effect of EVS; dots are observed mean SFDs at levels of EVS; **top right** : EVS × N+1 length interaction; **second row**, **left** : EVS x N-1 predictability; **second row**, **right** : EVS × N predictability; **third row** : EVS × N-frequency × N-1-frequency; **bottom row** : EVS × launch site × N-1 length. Factors in panels are based on median splits for visualization; LMM estimation used continuous covariates. Error bands represent 95% confidence intervals based on LMM residuals. Effects are plotted on a log-scale of fixation durations, thus they show the backtransformed effects as they were estimated in the LMM.

The analyses in the last section demonstrated that EVS at the end of a fixation (offset EVS) is strongly predictive of regressive and refixation saccades. In this section, we test whether fixation durations that are followed by a forward saccade are influenced by onset EVS. On the assumption that not only eye movements (i.e., regressions and refixations), but also fixation durations are in the service of maintaining fluent speech, the spatial EVS at fixation onset, should be predictive of the subsequent fixation duration. Specifically, the expectation is that if the EVS at fixation onset is large, long fixations should follow. There was clear evidence for this hypothesis in the data (see top left panel in **Figure 4**). The partial effect of onset-EVS on SFD (i.e., the regression line) represents a good fit of the observed mean SFDs at the various EVS levels (i.e., the dots). EVS at fixation onset was one of the strongest predictors of SFD, and had a substantial linear influence that was larger than well-established effects such as word frequency or word predictability.

The partial effect of EVS was estimated with statistical control of (a) the other covariates listed in **Table 3**, (b) differences between subject-related and sentence-related differences in mean fixation duration and effects, (c) subject-related and sentence-related differences in five effects each (i.e., variance components for N-length, N-frequency, N-predictability, N-1-length, and N-1-frequency effects, listed in **Table 4**), and (d) correlations between subject-related (-0.43) and sentence-related (+0.80) effects of length and frequency (i.e., correlation parameters). Estimates, standard errors, and *t*-values are reported in **Table 3**. We describe effects as significant if *t*-values are larger than 2.0. This is a conservative criterion because, given our past research, all statistical inference is one-tailed.

**Table 3:** Fixed-effect estimates of LMM for single fixation durations (SFDs), including EVS as covariate.

		Estimate	*SE*	*t-value*		Estimate	*SE*	*t-value*
Grand Mean SFD		5.492	0.018	**301.38**	Main effect of EVS	0.012	0.001	**14.68**
N-1 length	Linear	-0.170	0.988	-0.17	EVS × N-1 length	-0.021	0.138	-0.15
	Quadratic	0.771	0.478	-0.61		0.232	0.088	**2.65**
N-1 frequency	Linear	-0.689	0.868	-0.79	EVS × N-1 frequency	0.115	0.131	0.88
	Quadratic	-0.969	0.500	-1.94		0.050	0.078	0.64
N-1 predictability	Linear	0.559	0.440	1.27	EVS × N-1 predictability	-0.246	0.083	-**2.97**
	Quadratic	0.416	0.449	0.93		0.121	0.080	1.52
N length	Linear	5.006	1.081	**4.63**	EVS × N length	-0.204	0.133	-1.54
	Quadratic	0.342	0.441	0.78		0.070	0.078	0.90
N frequency	Linear	-0.138	1.214	-0.11	EVS × N frequency	-0.656	0.138	-**4.74**
	Quadratic	2.296	0.553	**4.15**		-0.159	0.096	-1.65
	Cubic	-2.668	0.500	-**5.34**		-0.025	0.091	-0.28
N predictability	Linear	-2.096	0.734	-**2.86**	EVS × N predictability	0.217	0.086	**2.53**
	Quadratic	1.487	0.470	**3.16**		-0.142	0.078	-1.82
N+1 length	Linear	0.166	0.708	0.24	EVS × N+1 length	-0.312	0.132	-**2.36**
	Quadratic	-2.012	0.457	-**4.40**		0.064	0.081	0.80
N+1 frequency	Linear	-2.350	0.738	-**3.18**	EVS × N+1 frequency	-0.167	0.138	-1.22
	Quadratic	0.409	0.461	0.89		-0.045	0.085	-0.53
N+1 predictability	Linear	1.055	0.466	**2.27**	EVS × N+1 predictability	-0.113	0.080	-1.42
	Quadratic	0.843	0.441	1.91		-0.020	0.075	-0.27
launch site distance	Linear	6.525	0.388	**16.80**	EVS × launch site distance	-0.152	0.083	-1.83
	Quadratic	1.099	0.306	**3.59**		0.037	0.067	0.55
landing site	Linear	6.137	0.370	**16.60**	EVS × landing site	0.015	0.083	0.18
	Quadratic	-0.423	0.309	-1.37		-0.024	0.072	-0.34
saccade size	Linear	7.542	0.363	**20.78**	EVS × saccade size	0.155	0.080	1.93
	Quadratic	1.971	0.313	**6.31**		0.090	0.067	1.35
N- freq x N-1 freq	Linear	3.258	0.325	**10.02**	EVS × N- frequency × N-1 freq	0.116	0.057	**2.05**
	Quadratic	1.227	0.329	**3.73**		-0.048	0.055	-0.88
	Cubic	-1.252	0.318	-**3.94**		-0.013	0.057	-0.23
N-freq x N+1 freq	Linear	0.880	0.325	**2.71**	EVS × N-frequency × N+1 frequency	-0.094	0.061	-1.54
	Quadratic	0.304	0.358	0.85		-0.105	0.064	-1.64
	Cubic	0.055	0.380	0.14		0.035	0.068	0.52
N-1 length x launch site distance		-0.076	0.012	-**6.14**	EVS × N-1 length x launch site distance	-0.007	0.003	-**2.29**

*Eye-voice span was specified as a centered covariate. Therefore, the intercept estimates the Grand Mean SFD. Main effects of covariates (and associated test statistics) are presented in the left four columns; coefficients for their interactions with EVS in the right four columns; see text for details. Bold values indicate significant contrasts.*

**Table 4:** Variance components and correlation parameters for LMMs.

EVS LMM			Oral/Silent LMM		
Random factor	Variance component	*SD*	Random factor	Variance component	*SD*
Sentence	N-length	0.177	Sentence	N-length	0.185
(*N* = 144)	N-frequency	0.052	(*N* = 144)	N-frequency	0.049
	N-predictability	0.171		N-predictability	0.192
	N-1 frequency	0.024		N-1 frequency	0.032
	N-1 length	0.092		N-1 length	0.105
	Mean SFD	0.062		Mean SFD	0.045
Subject	N-length	0.098	Subject	N-length	0.081
(*N* = 32)	N-frequency	0.029	(*N* = 63)	N-frequency	0.023
	N-predictability	0.055		N-predictability	0.041
	N-1 frequency	0.012		N-1 frequency	0.010
	N-1 length	0.078		N-1 length	0.075
	Mean SFD	0.096		Mean SFD	0.114
Residual (*N* = 11709)		0.272	Residual (*N* = 31185)		

*Correlation parameters for were 0.80 and -0.40 for sentence-related and subject-related N-length and N-frequency effects, respectively, in the EVS LMM; corresponding correlation parameters were 0.82 and -0.55 in the oral/silent LMM.*

The main EVS effect was moderated by (interacted with) length of the next word N+1 (i.e., N+1-length), N-frequency, N-predictability, and N-1-predictability. In addition, there were two three-covariate interactions: EVS × N-frequency × N-1-frequency and EVS × N-1-length × launch distance (see **Table 2**). These interactions are shown in the remaining panels of **Figure 4**.

##### EVS × N+1-length

An effect of the length of the next word is obtained for short EVS. Presumably, with short EVS weight of processing can shift in the direction of reading, increasing the chances of observing a parafoveal-on-foveal effect of word length.

##### EVS × N-1-predictability

If the last word was of low predictability the EVS slope was steeper than when the last word was highly predictable. High processing difficulty appears to be associated with stronger EVS effects.

##### EVS × N predictability

An effect of the predictability of the fixated word is obtained for short onset-EVS, but not for long onset-EVS. This suggests that if the voice lags far behind the eye at fixation onset, prediction of the fixated word is limited. It can possibly be interpreted as a working memory effect; if the working memory buffer is too full, prediction of the upcoming word becomes very hard.

##### EVS × N-frequency × N-1-frequency

The third row of **Figure 4** displays the interaction between current and last-word frequency for small and large EVS. This interaction also subsumes the EVS × N-1-frequency interaction. The most striking feature is the high-N-frequency hump after high frequency words N-1. This two-way interaction (also in its direction) was already reported in Kliegl et al. (2006; also Kliegl, 2007). The most plausible interpretation is that it reflects processing of word N+1 during a fixation on word N. We suggest that the attenuation of the high-frequency hump when word N-1 was of low frequency is evidence for less parafoveal processing during these fixations, presumably due to needs to deal with spillover from the last word. Qualitatively, this interaction was similar for short and large EVS. With a focus on differences, frequency effects were larger and more linear when the EVS was large. EVS moderated the frequency effect on fixation durations even more strongly when word N-1 was of low frequency; a strong and more or less linear N-frequency effect was observed in this case when EVS was large, whereas the N-frequency effect had little time to unfold when EVS was small. Thus when the onset-EVS is large, more cognitive resources seem to be allocated to processing of the current word rather than its neighbors.

##### EVS × N-1-length × launch site

The fourth row of **Figure 4** displays the interaction between launch site and length of word N-1 for small and large EVS. Fixation durations are especially long for the combination of large launch site and short words. Presumably the major source of this interaction is skipping which, on the one hand, is strongly linked to short words and, on the other hand, it is commonly accepted that fixations after skipped words are longer than average (e.g., Kliegl and Engbert, 2005, Table 1 for a review). Again, this interaction was qualitatively similar for short and large EVS. In this case, the effect of EVS for short last words was larger for long launch sites (i.e., high skipping probability).

### Distributed Processing during Oral Reading

Fixation durations are not only predicted by the EVS, but also sensitive to numerous visual and lexical indicators of processing difficulty as well as to oculomotor demands. All the covariates listed in **Table 3** were used in previous research on silent reading and almost all of them showed consistent effects across nine samples of readers (e.g., Kliegl et al., 2006; Kliegl, 2007). In the previous section we used these variables as statistical control variables for assessing the effect of onset EVS on fixation duration. In this section, we assess their effects on their own right, so to say, by comparing them directly with a group of readers who read the same sentences silently. With one exception, this second LMM was identical to the first LMM reported above. Instead of the within-subject covariate EVS, we included the between-subject variable oral vs. silent reading. Estimates, standard errors, and *t*-values for the second LMM are reported in **Table 5** ; estimates of variance components are listed in **Table 4**. Again, we describe effects as significant if *t*-values are larger than 2.0. Please note that, as this is an article about the EVS, there is not enough space to discuss in detail effects that relate to other domains of research on eye-movement control during reading. Therefore, this section will be selective in highlighting results that are likely to be of interest beyond the EVS context of eye-movement control during reading.

**Table 5:** Fixed-effect estimates of LMM for SFDs, comparing silent and oral reading.

		Estimate	*SE*	*t-value*		Estimate	*SE*	*t-value*
Mean oral SFD		5.514	0.021	**266.02**	Δ (s – o SFD)	-0.221	0.029	-**7.62**
N-1 length	Linear	-0.812	1.365	-0.60	Δ (s – o) N-1-length	5.565	1.622	**3.43**
	Quadratic	1.874	0.599	**3.13**		-0.613	0.648	-0.95
N-1 frequency	Linear	-0.340	1.177	-0.29	Δ (s – o)-N-1 frequency	3.613	1.169	**3.09**
	Quadratic	-2.683	0.627	-**4.28**		2.537	0.645	**3.94**
N-1 predictability	Linear	0.601	0.554	1.09	Δ (s – o)-N-1 predictor	-0.767	0.649	-1.18
	Quadratic	0.388	0.556	0.70		0.083	0.628	0.13
N length	Linear	7.583	1.564	**4.85**	Δ (s – o)-N length	-4.165	1.597	-**2.61**
	Quadratic	-0.190	0.613	-0.31		2.011	0.672	**2.99**
N frequency	Linear	-0.897	1.617	-0.55	Δ (s – o)-) N frequency	2.284	1.648	1.39
	Quadratic	5.156	0.701	**7.36**		0.098	0.737	0.13
	Cubic	-2.667	0.663	-**4.02**		1.478	0.682	**2.17**
N predictability	Linear	-2.970	1.052	-**2.82**	Δ (s – o)-N predictor	0.071	0.807	0.09
	Quadratic	1.760	0.602	**2.93**		-0.041	0.617	-0.07
N+1 length	Linear	0.954	0.900	1.06	Δ (s – o)- N+1 length	-0.757	1.021	-0.74
	Quadratic	-2.717	0.571	-**4.76**		1.657	0.629	**2.63**
N+1 frequency	Linear	-3.244	0.944	-**3.44**	Δ (s – o)-N+1 frequency	-1.215	1.061	-1.15
	Quadratic	0.631	0.576	1.10		-0.926	0.649	-1.43
N+1 predictability	Linear	1.138	0.568	**2.01**	Δ (s – o)-N+1 predictor	1.587	0.627	**2.53**
	Quadratic	1.710	0.554	**3.09**		0.122	0.606	0.20
launch site distance	Linear	11.128	0.546	**20.40**	Δ (s – o)-launch site distance	3.475	1.075	**3.23**
	Quadratic	1.965	0.436	**4.51**		1.694	0.883	1.92
landing site	Linear	11.547	0.623	**18.53**	Δ (s – o)-landing site	-4.571	0.994	-**4.60**
	Quadratic	-1.803	0.532	-**3.39**		-2.259	0.858	-**2.63**
saccade size	Linear	12.684	0.552	**22.97**	Δ (s – o)-saccade size	-8.372	0.789	-**10.61**
	Quadratic	2.484	0.421	**5.90**		0.690	0.684	1.01
N-frequency × N-1 freq	Linear	3.962	0.444	**8.93**	Δ (s – o)-N- frequency × N-1 frequency	-1.289	0.464	-**2.78**
	Quadratic	1.650	0.440	**3.75**		-0.250	0.442	-0.57
	Cubic	-1.121	0.439	-**2.55**		0.444	0.449	0.99
N-frequency × N+1 freq	Linear	1.461	0.431	**3.39**	Δ (s – o)- N-frequency × N+1 frequency	-0.705	0.462	-1.53
	Quadratic	1.234	0.484	**2.55**		-0.068	0.508	-0.13
	Cubic	0.561	0.490	1.15		-0.543	0.514	-1.06
N-1 length × launch site distance		-0.075	0.011	-**6.67**	Δ (s – o)- N-1 length × launch site distance	-0.070	0.019	-**3.75**

Reading condition was specified as a treatment contrast with oral reading as reference. Therefore, main-effect coefficients in the left four columns represent mean and covariate effects (slopes) for oral reading; coefficients in the right four columns represent corresponding differences between oral and silent conditions (i.e., interactions between reading condition and covariate; differences in slopes between conditions). Thus, the sum of corresponding coefficients yields the effects for silent reading. Example: D (s – o) N-1-length: partial-effect estimate of difference between silent and oral condition for slopes associated with length of last word. Bold values indicate significant contrasts.

#### Canonical Effects

**FIGURE 5:**
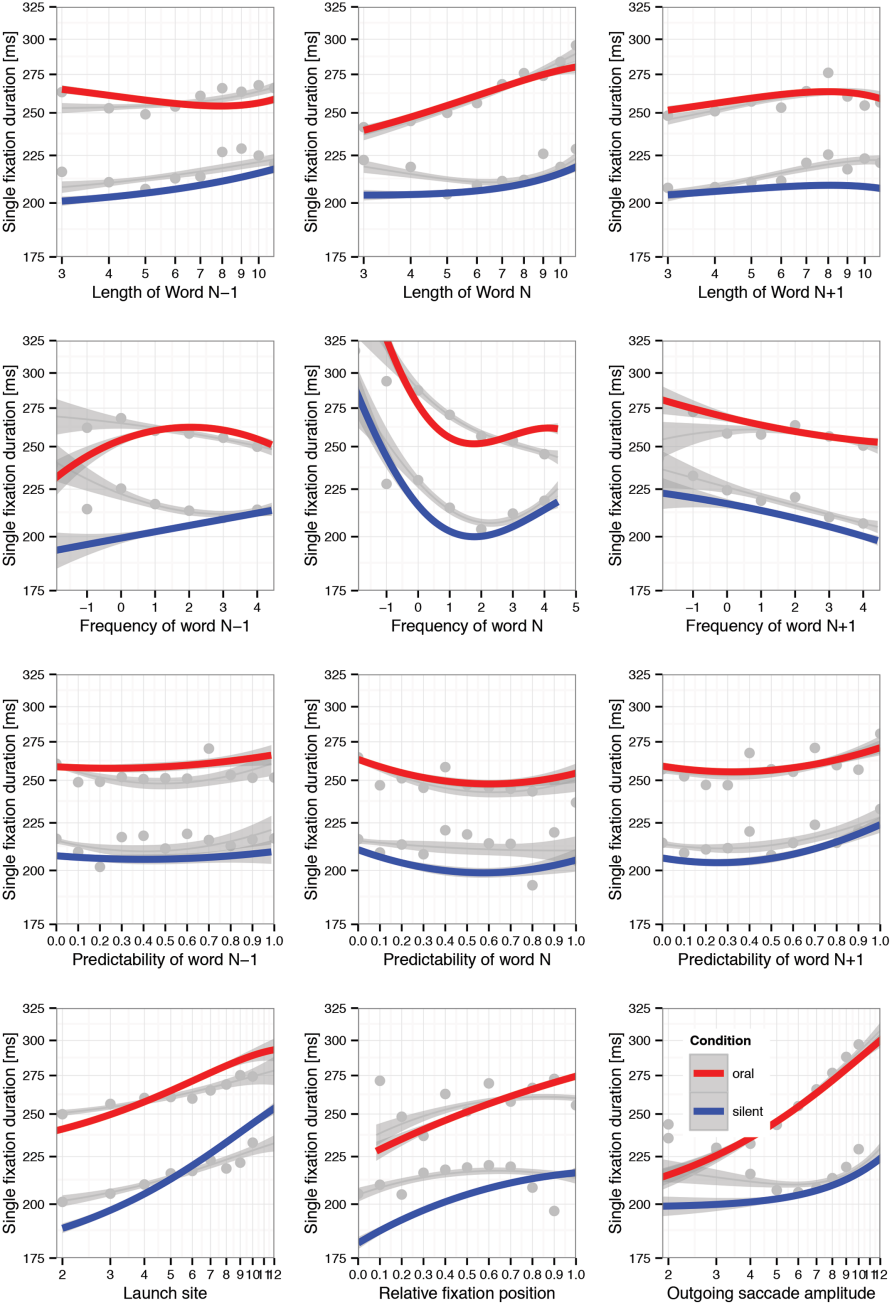
**Visualization of LMM estimates of interactions of reading condition (oral vs. silent) with 12 covariates.** Colored lines represent partial effects; gray lines represent zero-order effects (i.e., simple regression of SFD on covariate); dots are observed mean SFDs suitably binned for the specific covariate; error bands represent 95% confidence intervals based on LMM residuals. The interactions of reading condition with N-1-frequency, N-frequency, N-1-length, and launch site distance should not be interpreted as such, because they are subordinated to higher-order interactions (see **Figure 6**). Note that effects are plotted on a log-scale of fixation durations.

Effects of word length, frequency, and predictability of the fixated word, corresponding effects of its left and right neighbor as well as effects of launch site, fixation position within word, and the amplitude of the outgoing saccade count among the best-studied covariates for single-fixation duration during silent reading. **Figure 5** is modeled on Figure 3 of Kliegl et al. (2006), but displays partial effects both for silent (red lines) and oral (blue lines) reading (i.e., the interaction of reading condition with each covariate). In addition, the gray lines and gray dots in each panel inform about the zero-order (i.e., simple) regression of SFD on the covariates and observed means categorized according to some covariate-dependent binning. Those panels in which the red and blue lines depart substantially from their gray-line neighbors were much affected by statistical control.

Obviously, aside from the generally longer fixation durations during oral than silent reading, there is much similarity with respect to the direction and profile of the canonical effects. In general, fixation durations increased when processing was difficult. The direction and shape of well-established effects of word length, frequency, and predictability were similar in oral and in silent reading. However, there were also some differences between reading modes, which we will discuss further below.

#### Controversial and Novel Effects

Aside from corroboration of well-established effects, the data also provided new information on controversial effects. An in-depth discussion of each topic is beyond the scope of this article. Moreover, the results attest to the reliability of effects, but do not really lead to resolution of the associated theoretical controversies. Therefore, the report of these results is to serve primarily as a pointer to the relevant literature. All effects are shown in panels of **Figure 5**.

##### N+1-frequency and N+1-predictability

There were two controversial effects that were replicated quite strongly in both oral and silent reading: negative N+1-frequency effect and positive N+1 predictability effect. The direction of the former effect is canonical (i.e., shorter fixation durations for high N+1 frequency words) whereas the direction the latter is non-canonical (i.e., longer fixation durations for high N+1 predictability words. The opposite direction of effects on fixation duration is remarkable, given that frequency and predictability of words are positively correlated. Both effects were reported in Kliegl et al. (2006), but are not well understood, and evidence has primarily been obtained from corpus studies (Kennedy and Pynte, 2005; Kliegl, 2007; Rayner et al., 2007; Angele et al., 2015). Their appearance during oral reading strongly supports their reliability and may provide new perspectives on their explanation. First note that there is no statistical difference between oral and silent reading with respect to the negative N+1 frequency effect. Thus, this effect replicates across reading modes and with new sentence material. It likely indicates parafoveal preprocessing of the upcoming words. Second, the non-canonical positive N+1 predictability effect has been interpreted as an effect of memory retrieval (i.e., not as a parafoveal-on-foveal effect; Kliegl et al., 2006). Again the effect replicated across reading modes, although it also interacted with reading mode, as will be discussed below.

##### Fixation position

The signature effect of fixation position in word on SFD is the inverted u-shape of the function (Vitu et al., 2001). Again, several explanations have been advanced for this result (Nuthmann et al., 2005, for a review), including fast correction of mislocated fixations near the word boundaries. Our results reveal an important difference between the zero-order relation and the partial effects. The zero-order functions reveal a peak of SFDs in the word whereas for partial effects SFDs increase across the word. Note that all curves are of negative quadratic shape. The divergence between zero-order and partial effects suggests that the commonly observed decrease of SFDs toward the end of words is accounted for by covariates in the LMM. Most importantly, the result was obtained for the group of oral and the group of silent readers, despite minor differences, as will be discussed below.

##### N-1 frequency

**FIGURE 6:**
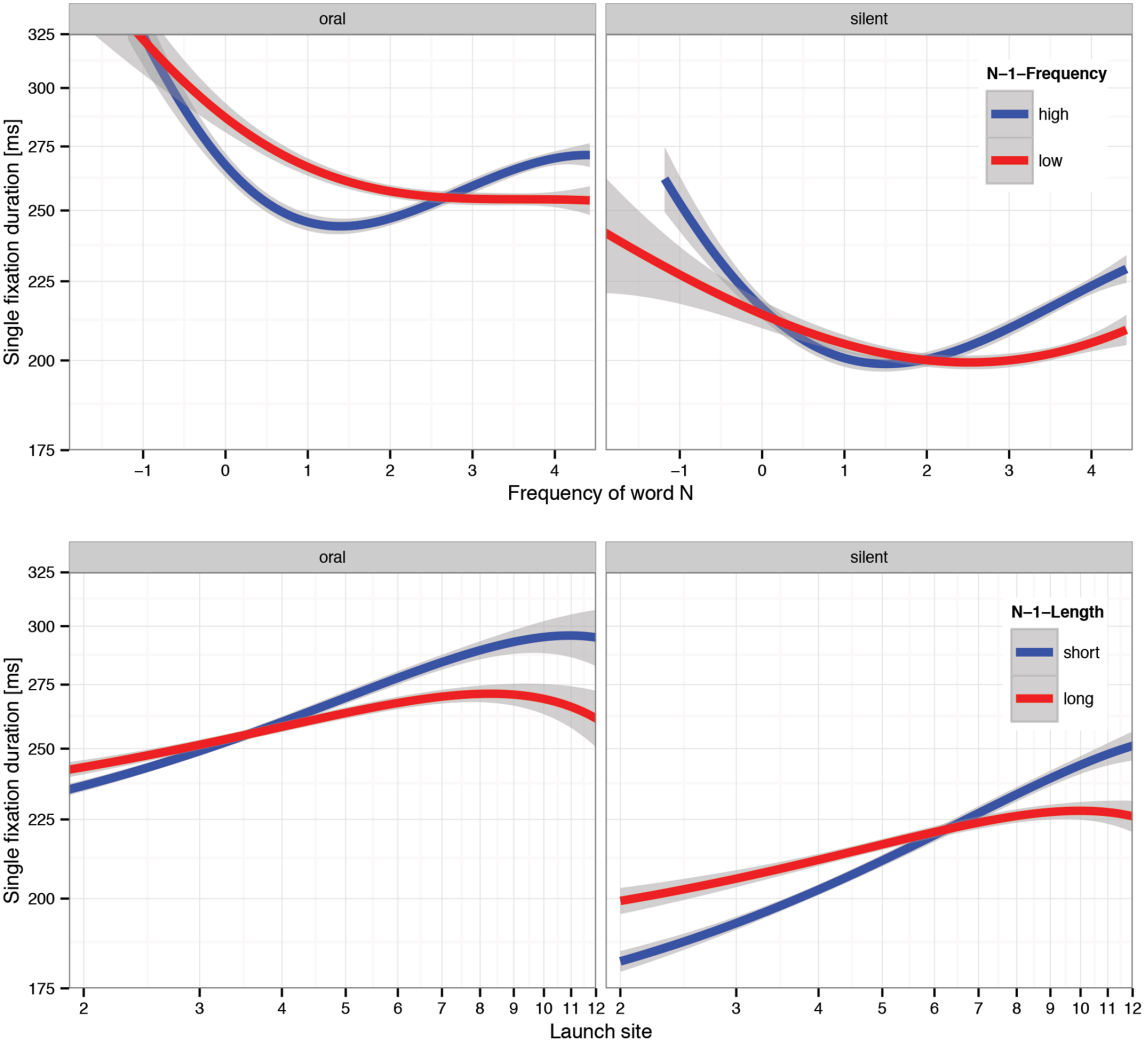
**Visualization of two LMM interactions involving three covariates; except reading condition, LMM used continuous covariates. Top row** : oral vs. silent × N-frequency × N-1 frequency. **Bottom row** : oral vs. silent × launch site × N-1 length. Factors in panels are based on median splits; LMM estimation used continuous covariates. Error bands represent 95% confidence intervals based on LMM residuals. Note that effects are plotted on a log-scale of fixation durations.

The second example of a strong and quite unexpected difference between zero-order and partial effects concerns the effect of the frequency of the last word. The zero-order functions exhibit the negative effect known from past research (e.g., Kliegl et al., 2006) for both oral and silent reading. Usually this pattern is interpreted as evidence for spillover from processing the previous word. In this case, the partial effects for the reading condition × N-1-frequency interactions are actually quite misleading and should not be interpreted because this interaction is subordinated to the three-covariate interaction reading-condition × N-1-frequency × N-frequency, shown in **Figure 6** (top row) and discussed below.

#### Evidence for Differences between Oral and Silent Reading

The LMM provides test-statistics for the interaction between reading condition and each of the covariates. This interaction was significant for 9 of 12 covariates (see **Table 5**). Four of them were nested within a higher-order interaction and will be covered in this context (for sake of completeness all two-way interactions with reading mode are visualized in **Figure 5**). Others are due to a quantitative rather than qualitative change in the degree of non-linearity. For example, the negative cubic trend of word-N frequency was present in both reading modes, but more pronounced in oral (-2.667) than in silent reading (-2.667 + 1.478 = -1.189). We had no specific expectation with respect to these differences; they were beyond the level of the current theoretical discourse. In the following we provide separate descriptions of these differences before an attempt at an integrative discussion.

##### Oral/silent main effect

As expected, silent reading was faster than oral reading. This at least partly reflects the need to wait for the slower voice, because otherwise working memory demands would become to great.

##### Oral/silent × N length

There were positive linear and quadratic effects for silent reading, but only a (stronger) positive linear effect for oral reading, suggesting that the whole range of word lengths affects SFD in oral reading, whereas the word length effect is restricted to longer words in silent reading.

##### Oral/silent × N+1 length

There were negative quadratic effect for both reading modes, which were stronger for oral reading.

##### Oral/silent × N+1 predictability

Positive linear and quadratic trends were observed in both reading modes; however, the linear component was stronger in silent reading. Since the effect of N+1 predictability has been linked to memory retrieval (Kliegl et al., 2006), this possibly indicates greater interference of ongoing articulatory planning with retrieval of expected words during oral reading.

##### Oral/silent × landing site

Although there were strong positive linear and negative quadratic effects for both modes, the linear trend was stronger and the quadratic trend weaker in oral reading. We had no particular expectations about reading mode differences in landing position. The IOVP-effect in silent reading has been linked to fast correction of mislocated fixations; it is possible that the oral reading constraint to maintain the EVS leads to a weaker influence of such lower-level oculomotor control mechanisms.

##### Oral/silent × saccade amplitude

The most striking interaction with reading condition involved the outgoing saccade amplitude (see **Figure 5**, bottom right panel). There was a much stronger increase in SFD with the amplitude of the next saccade for oral than for silent reading. This interaction might be related to EVS: if a reader plans a long saccade, possibly involving skipping of the next word, and if at the same time aim the EVS must not become too large, one option (or even a necessity) is to wait a little longer.

##### Oral/silent × N-1 frequeny × N frequency

Positive quadratic and negative cubic effects of word-N frequency were observed in both reading modes, but the latter was even stronger negative in oral reading. The quadratic trend, i.e., the upswing for the combination of high-frequency words N and high-frequency words N-1, indicates preprocessing of the upcoming word; there is increased parafoveal preprocessing when foveal processing is easy (Henderson and Ferreira, 1990; Kliegl et al., 2006). Since the cubic effect mainly dampens the upswing caused by the quadratic effect, this is possibly related to the somewhat smaller perceptual span in oral reading. However, when word N received less preprocessing due to a difficult word N-1, frequency effects were monotonous across the whole range. This effect was even stronger during oral reading, when low-frequency words N-1 are also associated with articulatory difficulty. In support of this interpretation, this effect in oral reading appears to be linked to a large EVS (see **Figure 4**, third row).

##### Oral/silent × launch site × N-1-length

There was also a very strong interaction between reading condition, launch site distance and length of the last word (see **Figure 6**, bottom row), analogous to the interaction between short vs. large EVS and the latter two covariates. The main source of this interaction is the steeper positive slope of launch site for short words N-1 during silent reading. This result is mainly due to a higher probability of skipping during silent reading (see **Table 1**) coupled with the well-known longer fixation durations following skipped words (Kliegl and Engbert, 2005). Again it suggests that parafoveal preprocessing of word N took place in both modes, but was more effective in silent reading.

In summary, although there were some differences due to reading mode, the overall pattern of effects looked rather similar for oral and silent reading. Most of the differences are probably related to the faster pace of silent reading. Some of them (i.e., the stronger linear outgoing saccade amplitude effect) appear to be linked to maintenance of the EVS; other effects (the stronger linear launch site distance effect, and the weaker negative cubic trend in current word frequency effect in silent reading) appear to indicate more parafoveal preprocessing in silent than in oral reading. The more restricted effects of both previous word length and previous word frequency suggest that lagged processing plays less of a role in oral than in silent reading. However, when word N-1 is of low frequency, word-N frequency effects are stronger in oral than in silent reading, suggesting a role of articulatory processing–note that during a fixation on word N, it is typically word N-2 that is pronounced, hence word N-1 is prepared for articulation. Finally, there was also a reading mode difference in the effect of N+1 predictability, which is stronger in silent than in oral reading, possibly suggesting phonological interference with lexical retrieval. Clearly, more experimental work is needed to support these interpretations.

## Discussion

Oral reading is considerably slower than silent reading because of the demand to produce intelligible speech. In principle, longer fixation durations might offer a better chance to shift attention into the parafovea and thereby increase parafoveal aspects. The present results rather show that, despite some differences, eye movements during oral and silent reading are similar in many respects^2^. However, by analyzing the EVS, we have identified a previously unobserved, but very important regulatory influence on eye movements during reading. The present study is the first systematic investigation of how the spatial distance that the eye leads the voice regulates eye movement behavior. We have found the EVS to be predictive of regressions, refixations, and fixation durations. Indeed, effects of the EVS were among the strongest effects observed in the LMM analyses. Thus, the EVS during oral reading is a critical variable regulating eye movement behavior during reading. Given the documented effects of subvocalization on eye movements during silent reading, there is good reason to suspect that many of these influences are also at work during silent reading.

Before discussing the EVS in detail, we will focus on two methodological aspects that the present analyses brought forward. First, covariates of fixation durations typically exhibit substantial correlations (e.g., length and frequencies of word correlate around 0.70). Multivariate statistical tests of the significance of individual covariates take these correlations into account and yield partial effects. If covariates were uncorrelated, the direction and magnitude of the observed (zero-order) effect would be identical to the partial effect (i.e., there is no adjustment for uncorrelated covariates). With correlated covariates, in principle, there can be complete dissociation between zero-order and partial effects; Yan et al. (2014b) provide such dissociation for effects of word length and morphological complexity. In addition, in the presence of significant interactions between covariates, partial effects of the subordinate terms (i.e., the two main effects for a simple interaction) must not be interpreted independent of the interaction. The most striking example of this kind occurred for the N-1-frequency effects in oral and silent reading, which were nested under higher-order interactions involving N-frequency and reading condition.

Second, the LMMs were based on continuous covariates (except, naturally, the oral vs. silent reading condition). For visualization of interactions we binned one or two such covariates. Therefore, when interpreting interaction plots one must keep in mind that the visualization may have missed a major source of the interaction, perhaps apparent with a different, usually more fine-grained binning. Not withstanding this cautionary note, we are more impressed by the qualitative similarity of the interactions when comparing short or large EVS or when comparing oral and silent reading. In other words, as far as we can tell the significance of 3-covariate interactions are likely due to slight differences in the degree of non-linearity, not in the basic pattern. At this point such quantitative differences are clearly beyond the scope of theoretical proposals. Therefore, we primarily interpret the qualitatively similar interactions obtained across levels of EVS or across oral and silent reading as evidence for successful and non-trivial conceptual replications.

Returning to the EVS, the overall pattern of results suggests that the EVS is quite flexible, and is adjusted according to cognitive, oculomotor, and articulatory demands. Given that the voice proceeds fairly linearly through the text, most of the adjustment is actually performed by the oculomotor system. The eyes, and also the mind, could in principle proceed faster than the voice, since silent reading is faster than oral reading. However, the eyes need to wait for the voice because the size of the working memory buffer is limited. The major target value in the system controlling the eyes during oral reading is a constant EVS at fixation offset of about 10 letters, translating into an average temporal EVS of about 560 ms, in good agreement with Inhoff et al. (2011). The spatial EVS yielded a stronger signal for the dynamics than the temporal EVS, as suggested by the relatively narrow distribution of EVS at fixation offset compared to EVS at onset. This differentiation was much less pronounced for temporal EVS. There was also clear evidence that spatial offset-EVS is typically regulated within a fixation duration. Of course, sometimes this within-fixation adjustment fails and in these cases the probability of a refixation increases. If the EVS is too large for a refixation to effectively down-regulate the EVS, then a regression occurs with high probability.

It is worthwhile to put our results in a historical perspective. The absolute size of the onset-EVS is in surprisingly good agreement with Buswell’s early recordings, using Charles Judd’s sophisticated analog eye tracker with a tuning fork generating 50 Hz time stamps on a photo recording plate (Gray, 1917). In comparison, the EVS estimate from oﬄine studies using the lights-off paradigm (Levin and Buckler-Addis, 1979) is widely off-track, and while it might measure something useful, the label “EVS” is somewhat of a misnomer. We suspect that our on-line EVS method measures how much is typically buffered, i.e., how much potential buffering capacity is actually used, whereas the oﬄine method might measure its maximum under the most favorable circumstances. Why do the two estimates differ so widely? One reason could be the difference in tasks: whereas reading stops in the lights-off paradigm, it continues in the standard oral reading task, meaning that the working memory buffer needs to be continuously updated. Updating operations are costly and may be the reason for the much smaller estimate using the on-line measure.

Buswell furthermore reported that the EVS increased immediately prior to regressions, and was correlated with reading speed. Both of these results also hold in our data. Whereas Buswell had sophisticated recording equipment, he did not have any modern automated analysis tools or statistical models available. Thus, although he suspected that the EVS might be related to fixation duration, he was not able to find empirical evidence for this fact^3^, which was pronouncedly present in our data. Failing to find evidence for a modulation of fixation duration by the EVS, Buswell examined other potential causes for long fixations, and found that difficult words like “hypnagogic” or “hallucinations” caused increased fixation durations. In modern terms, he discovered a word frequency effect on fixation duration.

Returning to our results, we went beyond Buswell by showing that the frequency effect, which is now well documented for fixation durations, also interacts with the EVS, such that the regulation of the EVS by fixation duration is much stronger for low frequency words. We also found this regulatory effect to be stronger for low-predictability words to the left of the fixated word. This pattern seems best explained by an oculomotor strategy that is influenced by cognitive processing and allows the eye to scout further ahead only when there is free capacity in the working memory buffer. Finally, the anecdotal observation that the eye often scouts ahead when a sentence is initially revealed, followed by a regression to synchronize with the voice and to maintain a manageable buffer size, is also consistent with the hypothetical oculomotor strategy. In summary, the oculomotor system has several means to regulate the EVS at offset, e.g., adjustment of fixation duration, of saccade direction, and of saccade amplitude, and all of them appear to be used.

Reading aloud involves working memory, specifically the phonological loop. Indeed, due to the serial output requirement, the working memory buffer during reading aloud is in some respect akin to a first-in, first-out queue. Phonological information is stored in the buffer in the serial order needed for output, since rearranging the phonological buffer is quite difficult. However, it is not clear whether the corresponding lexical units are also serially activated. In fact, one major difference between current computational models of eye movement control during reading is whether they assume serial or parallel lexical activation.

What then are the implications of our results for reading models? Although the temporal and spatial parameters are slightly different from silent reading, the general pattern of effects on fixation durations and probabilities speak for a similar control mechanism in both reading modes. Therefore, current models for silent reading can be used as a starting point for models of oral reading. Arguably, one necessary extension is an on-line working memory buffer that operates during reading. In particular, our results provide strong evidence that the oculomotor system is regulated by the cognitive system such that a relatively constant amount of information is buffered in working memory. Critically, this buffer is constantly updated during reading, requiring on-line control. The control process regulates both where- and when-decisions of eye movements: a large EVS goes along with increases in fixation durations as well as refixation and regression probabilities. Our data thus provided temporal constraints for eye movement models, since it can probably be assumed that a word that has been articulated is no longer a member of the set of potential saccade target locations. In the SWIFT model, for example, the lexical activation of a word should again be at zero by the time the word is articulated. Although oral reading is somewhat slower than silent reading due to the output demand to produce comprehendible speech, the size of the working memory buffer during silent reading is probably limited as well; it might be somewhat larger, but is surely on the same order of magnitude, given that fixation durations are not that dramatically different and given that sub-vocalization also takes place during silent reading. Indeed, it may well be that oral-reading models do a better job of predicting performance in silent reading than the original models.

Modeling oral reading would thus be a worthwhile effort, and has implications far beyond eye movement control. At least in the U.S. and the UK, oral reading fluency is a major arena of reading instruction and a benchmark of educational success. In most of the education-related reading literature this is treated as a monolithic construct that is examined in relation to other equally abstract latent variables like “decoding” and “comprehension.” Research on the EVS has the potential to crack this black box open and begins to understand oral reading fluency in a much more fundamental way.

We presented a first description of the EVS, mainly using the approach of statistical control in multivariate analyses. Of course, further experimental analyses looking at specific aspects of the data will reveal new insights. In summary, we reported a detailed description of how during the EVS oral reading is regulated by cognitive processing difficulty. We discovered quite a few thought-provoking aspects of the cognitive regulation of the interplay between eye and voice during reading. The study provides an important first step at understanding how eye and voice are coordinated to achieve fast reading with a manageable working memory load.

## Conflict of Interest Statement

The authors declare that the research was conducted in the absence of any commercial or financial relationships that could be construed as a potential conflict of interest.

## Footnotes

1 Even with this computer-assisted procedure, scoring of word boundaries was rather laborious.

2 One difference between oral and silent reading is that oral reading requires the participant to retrieve and articulate each word accurately while silent reading requires only that the reader have extracted a sufficient understanding of the sentence to answer an easy multiple choice question. Skimming strategies are likely to be used under these low comprehension requirements. This difference is potentially heightened by the removal of oral reading trials with articulation errors – i.e., the same cannot be done for silent reading trials. It’s not necessarily clear how these selection effects would impact on the pattern of results, but it is unlikely to have had a major impact here, first, because of the overall similarity between the fixations duration patterns in oral and silent reading, and second, because of the relatively low number of sentences removed due to articulation errors.

3 This is probably a consequence of the fact that Buswell (1920, pp. 80f) only examined the span differences between the 10 longest and the 10 shortest fixations, and not at all of the data points.
